# The phospholipid-binding protein SESTD1 negatively regulates dendritic spine density by interfering with Rac1-Trio8 signaling pathway

**DOI:** 10.1038/srep13250

**Published:** 2015-08-14

**Authors:** Cheng-Che Lee, Chiung-Chun Huang, Kuei-Sen Hsu

**Affiliations:** 1 Department of Pharmacology, College of Medicine, National Cheng Kung University, Tainan, Taiwan

## Abstract

Dendritic spines are actin-rich protrusions from neuronal dendrites that harbor the majority of excitatory synapses. The balance of spine formation and retraction may influence dendritic integrity. While knowledge of the molecular mechanisms that promote dendritic spine formation has accumulated, little is known about the factors that limit spine formation. Here, we show that SESTD1, a phospholipid-binding protein containing a lipid-binding SEC14-like domain and two spectrin-repeat cytoskeleton interaction domains, negatively regulates dendritic spine density in cultured hippocampal neurons. Overexpression of SESTD1 decreases dendritic spine density in neurons by interfering with the interaction between Rac1 and its guanine nucleotide exchange factor (GEF) Trio8. Conversely, knockdown of SESTD1 increases dendritic spine density. Further analysis reveals that the SPEC1 domain-mediated interaction with Rac1 is required for SESTD1 activity toward a decrease in dendritic spine density. Transfection of GEF domain of Trio8 into neurons rescues SESTD1-mediated decrease in dendritic spine density. More importantly, overexpression of SESTD1 results in a decrease in the frequency of miniature excitatory postsynaptic currents (mEPSCs), whereas SESTD1 knockdown increases the mEPSC frequency. These results suggest that SESTD1 may act as a negative regulator of the Rac1-Trio8 signaling pathway to reduce dendritic spine density and lower excitatory synaptic transmission in hippocampal neurons.

Neurons communicate with each other via specialized structures called synapses that are composed of presynaptic and postsynaptic components. At excitatory synapses, the majority of synaptic input occurs at dendritic spines, which generally consist of a bulbous head and a thin neck connected to the dendritic shaft^1^,^2^. Dendritic spines show actin-based rapid motility, dynamic turnover and morphological plasticity^3^,^4^,^5^. Changes in the morphology and density of dendritic spines are believed to be crucial for maintaining synaptic function and plasticity^6^,^7^,^8^,^9^. Several plasticity-inducing stimuli can trigger *de novo* spine growth or elimination of pre-existing spines. In particular, the induction of long-term potentiation was found to correlate with an increase in spine growth^10^, whereas spine shrinkage occurred after long-term depression induction^11^,^12^. Moreover, the balance of spine formation and retraction may influence dendritic integrity^12^,^13^,^14^,^15^. Members of the Rho family GTPases, including RhoA, Rac1 and Cdc42, have been shown to play important roles in regulating spine dynamics by actin cytoskeleton rearrangement. For example, Rac1 and Cdc42 promote the development and maintenance of dendritic spines, whereas RhoA activation inhibits spine formation^5^,^16^,^17^. Recent work has mainly focused on determining the cellular and molecular mechanisms that promote dendritic spine formation and maintenance, but relatively little is known about the factors that limit dendritic spine formation.

SESTD1 (SEC14 and spectrin domains 1) is a recently cloned protein, originally identified as a binding partner of the transient receptor potential channels, TRPC4 and TRPC5^18^. It is highly expressed in many human tissues, including the brain, aorta, adipose and testis^18^. SESTD1 contains a SEC14-like lipid-binding domain and two spectrin-repeat domains (SPEC1 and SPEC2), which typically interact with the cytoskeleton. More recently, it has been reported that SESTD1 may cooperate with Dapper antagonist of catenin 1 (Dact1) scaffold protein to regulate the van Gogh-like protein 2 (Vangl2) and planar cell polarity pathway during embryonic development in mice^19^. Moreover, SESTD1 exhibits moderate sequence conservation with the Trio family proteins^20^, which may act as an early endosome-specific upstream activator of the Rho family GTPases for neurite elongation^21^. Based on these observations, we hypothesized that SESTD1 may regulate dendritic spine formation and thus affect synaptic function. Here, we show that SESTD1 is mostly located in the postsynaptic density of neurons, and negatively regulates dendritic spine density by interfering with the Rac1-Trio8 signaling pathway.

## Results

### Expression pattern of SESTD1 in rat hippocampus during development and embryonic hippocampal neurons in culture

**Figure 1:**
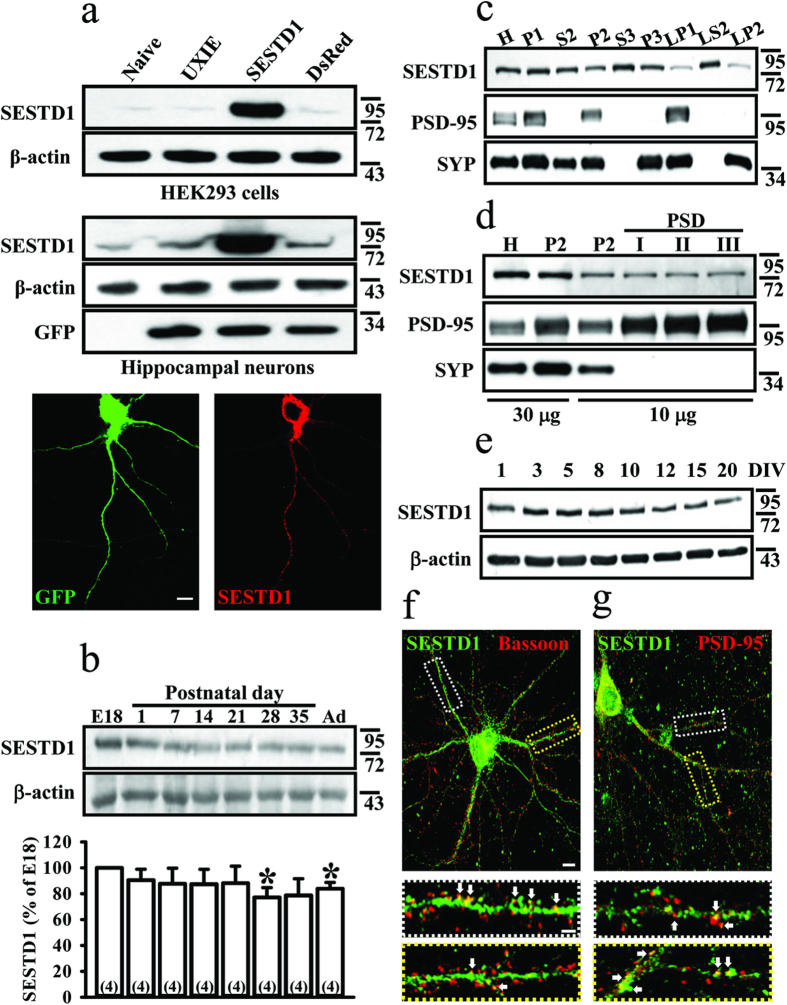
Expression pattern of SESTD1 in rat hippocampus and cultured hippocampal neurons. (**a**) Representative immunoblotting analysis showing the specificity of anti-SESTD1 antibody in the HEK293 cells and cultured rat hippocampal neurons overexpressing UXIE vector, DsRed or SESTD1, respectively. Immunostaining micrographs showing the validation of the specificity of the anti-SESTD1 antibody (red) in hippocampal neurons transduced with GFP-SESTD1 (green). Scale bar: 10 μm. (**b**) Representative immunoblotting and corresponding densitometric analysis showing the developmental expression of SESTD1 protein in embryonic day (E) 18, postnatal day (P) 1–35 and adult (Ad) rat hippocampal lysates. Statistical difference was compared with embryonic stage group. *p < 0.05; ANOVA with Bonferroni’s *post hoc* analyses from four independent experiments. (**c**) Representative immunoblotting analysis showing the subcellular localization of SESTD1 in P35 rat hippocampal lysates. H, homogenates; P1, nuclei and large debris; P2, crude synaptosomes; P3, light membranes; S2, supernatant after P2 precipitation; S3, cytosol; LP1, synaptosomal membranes; LP2, synaptic vesicle-enriched fraction; LS2, synaptosomal cytosol. Subcellular fractionations were confirmed by immunoblotting with anti-PSD-95 and synaptophysin antibodies, respectively. SYP: synaptophysin. (**d**) Representative immunoblotting analysis showing the expression of SESTD1 in postsynaptic density (PSD) fractions of P35 rat hippocampal lysates. Note that SESTD1 is existed in PSD fractions, extracted with Triton X-100 once (PSDI) or twice (PSDII), or with Triton X-100 plus sarkosyl (PSDIII). A total of 30 μg of H or P2, or 10 μg of P2 or PSD fraction samples was loaded in immunoblot experiments as indicated. (**e**) Representative immunoblotting analysis showing the expression of SESTD1 protein in cultured rat hippocampal neurons at 1–20 DIV. (**f**) Double immunostaining micrographs showing the distribution of SESTD1 (green) and presynaptic marker protein bassoon (red) in hippocampal neurons at 17 DIV. A higher magnification images (bottom panels) of region inside the white and yellow dotted line-boxes show the colocalization of immunostaining for SESTD1 and bassoon (arrows). (**g**) Double immunostaining micrographs showing the distribution of SESTD1 (green) and postsynaptic marker protein PSD-95 (red) in hippocampal neurons at 17 DIV. A higher magnification images (bottom panels) of region inside the white and yellow dotted line-boxes show the colocalization of immunostaining for SESTD1 and PSD-95 (arrows). Scale bars: 10 μm (top) and 2 μm (bottom).

To verify the specificity of the anti-SESTD1 antibody, Western blot analysis of lysates of HEK293 cells and cultured hippocampal neurons overexpressing SESTD1 was conducted. The anti-SESTD1 antibody specifically detected a band of approximately 79 kDa, consistent with the molecular weight of SESTD1 protein (Fig. 1a). In addition, the specificity of the anti-SESTD1 antibody was further confirmed by immunofluorescence staining of cultured hippocampal neurons transduced with GFP-tagged SESTD1 (Fig. 1a). Toward understanding the neuronal functions of SESTD1, we first determined the expression patterns of SESTD1 in the developing and adult rat hippocampus. SESTD1 protein expression in rat hippocampal tissue lysates was relatively high during the embryonic stage and the first postnatal week, and remained constant in adulthood (Fig. 1b). We further examined the expression of SESTD1 protein in various subcellular compartments of rat hippocampal tissues using subcellular fractionation method. The reliability of this method was confirmed by postsynaptic density protein-95 (PSD-95) and synaptophysin (SYP) as markers for the subcellular compartments as described previously^22^. As shown in Fig. 1C, SESTD1 was detected in the nuclei and large debris (P1), cytosol (S2, S3), synaptosomal cytosol (LS2), crude synaptosomes (P2), light membranes (P3), synaptosomal membranes (LP1) and synaptic vesicle-enriched fraction (LP2). In addition, SESTD1 was also found in the PSD fractions, in which they were resistant to extraction by Triton X-100 and sarkosyl detergents (Fig. 1d). We further confirmed that SESTD1 expression was high at the early developmental stages of primary hippocampal neurons cultured from E18 rat embryos and its expression levels remained high throughout neuronal maturation (Fig. 1e).

We also used immunolocalization with confocal fluorescence microscopy to qualitatively determine whether SESTD1 protein is present at the postsynaptic site. Immunoflurorescence staining of SESTD1 in mature hippocampal neurons at 17 days *in vitro* (DIV) revealed a patch- or cluster-like pattern. In addition, our double-staining studies showed that SESTD1 was distributed to synapses where it co-localized with the presynaptic cytomatrix protein bassoon (Fig. 1f) and the postsynaptic marker PSD-95 along dendrites in hippocampal neurons (Fig. 1g), indicating a synaptic localization of SESTD1 in hippocampal neurons.

### SESTD1 negatively regulates dendritic spine density

**Figure 2:**
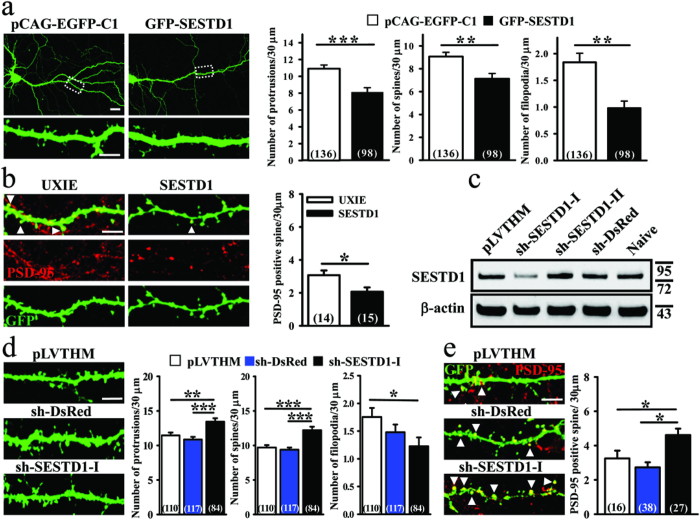
SESTD1 negatively regulates dendritic spine and filopodia density in hippocampal neurons. (**a**) Representative images of hippocampal neurons co-transduced with EGFP-β-actin plus pEGFP-C1 vector or GFP-SESTD1 (GFP fusion SESTD1 plasmid) at 12–13 DIV. Cells were fixed at 17 DIV and stained for GFP. Scale bars: 20 μm (top) and 5 μm (bottom). The bar graphs show the quantification of density of dendritic protrusion (spine and filopodia), spine, and filopodia (number/30 μm) of the representative groups. (**b**) Representative dendritic segments and quantification analysis showing the density of mature (PSD-95-positive) spine in UXIE vector- or SESTD1 (bicistronic expression plasmid)-transduced neurons at 17 DIV. Cells were stained for GFP and PSD-95. Scale bar: 5 μm. (**c**) Effective and specificity of SESTD1-shRNAs. Western blotting showing total lysates of hippocampal neurons transduced with pLVTHM vector, shRNA constructs targeting SESTD1 (sh-SESTD1-I and sh-SESTD1-II) and control shRNA targeting DsRed (sh-DsRed) at 17 DIV using antibodies against SESTD1 and β-actin, respectively. The level of SESTD1 was specifically reduced by sh-SESTD1-I construct. (**d**) Representative dendritic segments of hippocampal neurons transduced with pLVTHM vector, sh-DsRed or sh-SESTD1-I at 12–13 DIV. Cells were fixed at 17 DIV and stained for GFP. Scale bar: 5 μm. The bar graphs show the quantification of density of dendritic protrusion (spine and filopodia), spine and filopodia (number/30 μm) of the representative groups. (**e**) Representative dendritic segments and quantification analysis showing the density of mature (PSD-95-positive) spine in pLVTHM vector-, sh-DsRed or sh-SESTD1-I-transduced neurons at 17 DIV. Cells were stained for GFP and PSD-95. Scale bar, 5 μm. Numbers indicate the number of dendrites quantified. Statistical difference was compared with control vector group. *p < 0.05, **P < 0.01, ***p < 0.001; ANOVA with Bonferroni’s *post hoc* analyses.

Because dendritic spines represent the main unitary postsynaptic compartments for excitatory inputs^1^,^2^, the presence of SESTD1 at postsynaptic site and its expression pattern suggest that SESTD1 may regulate dendritic spine development. To examine this possibility, we overexpressed SESTD1 in cultured hippocampal neurons at 12–13 DIV and then examined the density of dendritic spine at 17 DIV. Hippocampal neurons were co-transduced with EGFP-β-actin encoding plasmid to help identify dendritic spines. We found that total protrusion (F_(1,233)_ = 19.5, p < 0.001), spine (F_(1,233)_ = 15.1, p < 0.001) and filopodia density (F_(1,233)_ = 10.2, p < 0.01) were significantly reduced compared with control pEGFP-C1 vector-transduced neurons (Fig. 2a). Because PSD-95 is a critical component of mature dendritic spines^23^, we therefore used PSD-95 immunohistochemistry to examine whether overexpressed SESTD1 also decreased mature dendritic spine density in hippocampal neurons. Immunostaining of endogenous PSD-95 in SESTD1 overexpression neurons showed a significant reduction in the number of PSD-95-positive puncta in dendrites compared with control UXIE vector-transduced neurons (F_(1,28)_ = 6.8, p < 0.05; Fig. 2b).

The importance of SESTD1 in the regulation of dendritic spine density was further determined by transducing hippocampal neurons with lentiviral construct encoding shRNA against SESTD1 at 12–13 DIV. Measurement of SESTD1 levels by Western blotting analysis revealed a significant reduction in the expression of SESTD1 protein in neurons transduced with SESTD1 shRNA-I (sh-SESTD1-I) but not sh-SESTD1-II compared with control pLVTHM vector or DsRed shRNA (sh-DsRed)-transduced neurons (Fig. 2c), confirming the effectiveness of shRNA to knockdown SESTD1. As shown in Fig. 2d, a significant increase in total protrusion (F_(2,308)_ = 10.1, p < 0.01) and spine density (F_(2,308)_ = 16.6, p < 0.001) was observed in neurons transduced with sh-SESTD1-I compared with control pLVTHM vector- or sh-DsRed-transduced neurons at 17 DIV. However, knockdown of SESTD1 did not change filopodia density (F_(1,200)_ = 2.2, p = 0.15) in neurons compared with sh-DsRed-transduced neurons, although a significance was observed in neurons between transduced with sh-SESTD1-I and control pLVTHM vector (F_(1,193)_ = 5.5, p < 0.05). Furthermore, transducing neurons with sh-SESTD1-I significantly increased the number of PSD-95-positive puncta in dendrites compared with control pLVTHM vector- or sh-DsRed-transduced neurons (F_(2,78)_ = 6.4, p < 0.05; Fig. 2e). These results suggest that SESTD1 is a negative regulator of dendritic spines.

### SESTD1 reduces dendritic spine density via its SPEC1 domain

**Figure 3:**
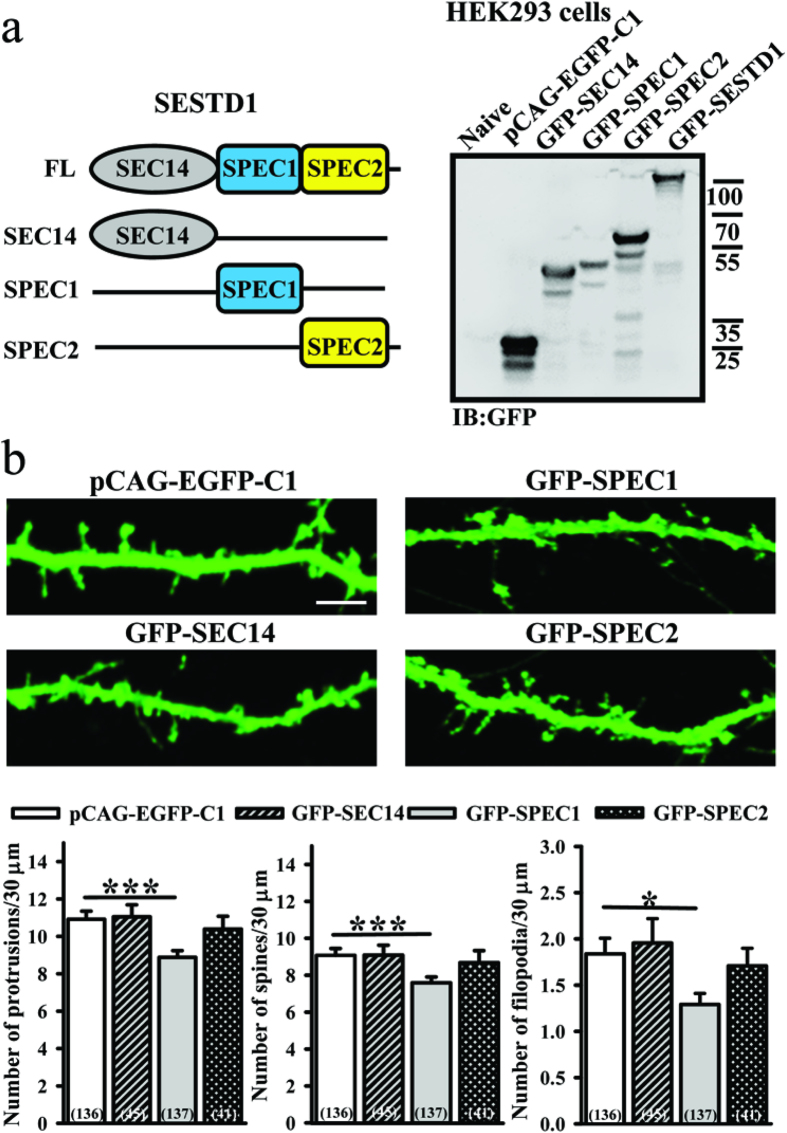
The SPEC-1 domain is important for SESTD1 to regulate dendritic spine and filopodia density in hippocampal neurons. (**a**) Schematics showing domains and deletion mutants of SESTD1. SPEC1, spectrin repeat domain 1; SPEC2, spectrin repeat domain 2. SESTD1 and various deletion mutants were expressed in HEK293 cells and cell lysates were immunoblotted with anti-GFP antibody. (**b**) Representative images of hippocampal neurons transduced with pCAG-EGFP-C1 vector, GFP-SEC14, GFP-SPEC1 or GFP-SPEC2 at 12–13 DIV. Cells were fixed at 17 DIV and stained for GFP. The bar graphs show the quantification of density of dendritic protrusion (spine and filopodia), spine, and filopodia (number/30 μm) of the representative groups. Scale bar: 5 μm. Numbers indicate the number of dendrites quantified. Statistical difference was compared with control pCAG-EGFP-C1 vector group. *p < 0.05, ***p < 0.001; ANOVA with Bonferroni’s *post hoc* analyses.

SESTD1 comprises multiple structural domains, including SEC14-like, SPEC1 and SPEC2^18^. To further identify the exact domain(s) on SESTD1 that undergoes negative regulation of dendritic spine density, we cloned three fragments of SESTD1 (SEC14, SPEC1 and SPEC2) and tagged with GFP. The full-length and fragments of SESTD1 were separately expressed in HEK293 cells and confirmed by Western blotting analysis using the anti-GFP antibody (Fig. 3a). We then co-transduced hippocampal neurons with β-actin-EGFP and construct encoding different truncated forms of SESTD1 at 12–13 DIV and examined the effects on dendritic spine density at 17 DIV. We found that transducing neurons with SPEC1 resulted in a significant reduction of total protrusion (F_(1,272)_ = 12.5, p < 0.001), spine (F_(1,272)_ = 10.9, p < 0.001) and filopodia density (F_(1,272)_ = 6.5, p < 0.05; Fig. 3b). However, transducing neurons with neither SEC14 nor SPEC2 significantly altered the density of total protrusion, spine or filopodia. These results suggest that SPEC1 domain is necessary and sufficient for the SESTD1-mediated decrease in dendritic spine density.

### SESTD1 reduces dendritic spine density through the suppression of Rac1 activity

**Figure 4:**
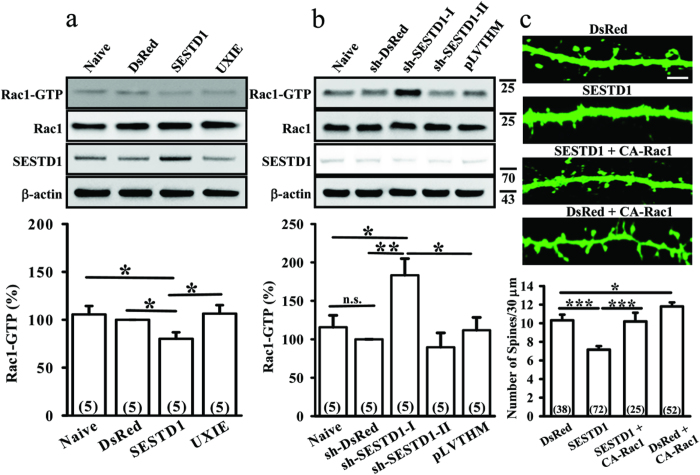
SESTD1 decreases dendritic spine density by suppressing Rac1 activity. (**a**) Representative immunoblots and corresponding densitometric analysis showing the levels of Rac1-GTP (Rac1 activity) in naive, UXIE vector-, DsRed- or SESTD1-transfected neurons at 17 DIV. (**b**) Representative immunoblots and corresponding densitometric analysis showing the levels of Rac1-GTP in naive, pLVTHM vector-, sh-DsRed-, sh-SESTD1-I or sh-SESTD1-II-transduced neurons at 17 DIV. *p < 0.05, **p < 0.01; ANOVA with Bonferroni’s *post hoc* analyses from five independent experiments. (**c**) Representative dendritic segments and quantification analysis showing dendritic spine density in DsRed-, SESTD1, SESTD1 plus constitutively active Rac1 (CA-Rac1)- or DsRed- plus CA-Rac1-transfected neurons at 17 DIV. Scale bar: 5 μm. Numbers indicate the number of dendrites quantified. *p < 0.05, ***p < 0.001; ANOVA with Bonferroni’s *post hoc* analyses.

Given that Rac1, a member of the Rho family GTPases, is functionally crucial in maintaining the morphology and density of dendritic spines^24^,^25^,^26^ and that loss-of-function mutation of Rac1 results in decreased dendritic spine density^16^, we tested whether SESTD1 elicits its inhibitory effect on dendritic spine density via an inhibition of Rac1 activity. We found that overexpressed SESTD1 significantly reduced the amount of active GTP-bound Rac1 (Rac1-GTP) in hippocampal neurons compared with naive (F_(1,9)_ = 5.3, p < 0.05), control UXIE vector (F_(1,9)_ = 5.7, p < 0.05) or DsRed-transduced neurons (F_(1,9)_ = 8.7, p < 0.05; Fig. 4a). In contrast, a significant increase in the amount of Rac1-GTP was observed in neurons transduced with sh-SESTD1-I but not sh-SESTD1-II compared with naive (F_(1,9)_ = 6.6, p < 0.05), control pLVTHM vector- (F_(1,9)_ = 5.96, p < 0.05) or sh-DsRed-transduced neurons (F_(1,9)_ = 14.9, p < 0.01; Fig. 4b). There were no significant differences in the total amount of Rac1 expression between neurons transduced with UXIE, DsRed, SESTD1, pLVTHM, sh-DsRed, sh-SESTD1-I or sh-SESTD1-II.

We then investigated whether restoration of Rac1 activity may rescue dendritic spine loss in SESTD1 overexpressing neurons. As shown in Fig. 4c, expression of a constitutively active form of Rac1 mutant (Q61L; CA-Rac1) in SESTD1 overexpressing neurons fully restored normal dendritic spine density (F_(1,96)_ = 12.6, p < 0.001). These results suggest that the reduction of Rac1 activity is a critical downstream molecular event of SESTD1-mediated decrease in dendritic spine density.

### SESTD1 interferes with the binding of Trio8 to Rac1

**Figure 5:**
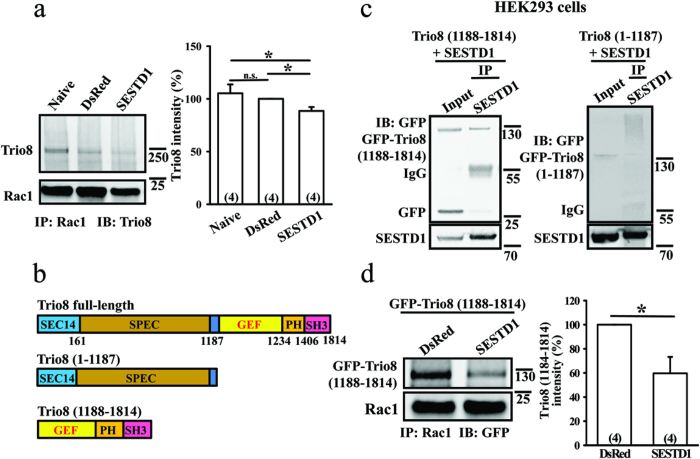
SESTD1 interferes with the binding of Trio8 to Rac1. (**a**) Co-immunoprecipitation and immunoblot analysis of the interaction between Rac1 and Trio8. Note that overexpressed SESTD1 significantly reduced the interaction between Trio8 and Rac1 in hippocampal neurons at 17 DIV. *p < 0.05; ANOVA with Bonferroni’s *post hoc* analyses from four independent experiments. n.s., not significant. (**b**) Schematics representation of full-length and two deletion mutants of Trio8. SPEC, spectrin repeat domain; GEF, guanine nucleotide exchange factor; PH, pleckstrin homology; SH3, Src homology 3. The deletion mutant Trio8 (1–1187) consists SEC14 and SPEC domains, whereas Trio (1188–1814) consists GEF, PH and SH3 domains. (**c**) The GFP-tagged deletion mutants of Trio8 were co-transduced with SESTD1 in HEK293 cells. Cell lysates were immunoprecipitated with antibody to SESTD1 or IgG and then immunoblotted with GFP antibody. (**d**) Overexpressed SESTD1 significantly reduced the interaction between Trio8 (1188–1814) and Rac1 in hippocampal neurons at 17 DIV. Cell lysates were immunoprecipitated with antibody to Rac1 and then immunoblotted with anti-GFP antibody. *p < 0.05; ANOVA with Bonferroni’s *post hoc* analyses from four independent experiments.

SESTD1 shows sequence similarity with Trio family members in its N-terminal spectrin-like domains^20^, but it lacks a guanine nucleotide exchange factor (GEF) domain typically required for activating Rho family GTPases. Solo/Trio8 is a membrane-associated short isoform of Trio and may act as an early-endosome-specific upstream activator of Rac1 for neurite elongation of developing Purkinje neurons^21^. It was temping to speculate that SESTD1 may regulate Rac1 activation and dendritic spine density by interfering with the binding of Trio8 to Rac1. To test this possibility, we examined their physical interaction in hippocampal neurons and found that Trio8 coimmunoprecipitated with Rac1. Indeed, immunoprecipitation of cell lysates with the Rac1 antibody resulted in the detection of Trio8 protein (Fig. 5a). Furthermore, compared with naive or DsRed-expressing neurons, overexpressed SESTD1 significantly reduced coimmunoprecipitation of Trio8 with Rac1 (p < 0.05), indicating that SESTD1 inhibits recruitment of Trio8 to activate Rac1.

Trio8 comprises multiple structural domains, including SEC14, SPEC, GEF, pleckstrin homology (PH) and Src homology 3 (SH3)^21^. To determine whether the GEF domain of Trio8 activates Rac1, we cloned two fragments of Trio8 [Trio8 (1–1187) and Trio8 (1188–1814)] and tagged with GFP (Fig. 5b). The fragments of Trio8 proteins were co-transduced with SESTD1 in HEK293 cells and protein complexes were analyzed by coimmunoprecipitation (Fig. 5c). Interestingly, we found that Trio8 (1188–814) lacking N-terminal residues, including SEC14 domain and SPEC domains, can form complexes with SESTD1. In addition, compared with DsRed expressing cells, overexpression of SESTD1 significantly reduced coimmunoprecipitation of Trio8 (1188–1814) with Rac1 (F_(1,7)_ = 8.6, p < 0.05; Fig. 5d). In contrast, Trio8 (1–1187) fragment is insufficient to mediate complex formation with Rac1 or SESTD1 (Fig. 5c). These coimmunoprecipitation data suggest that the C-terminal residues of Trio8, including GEF, PH, and SH3 domains, are required for complex formation with Rac1.

**Figure 6:**
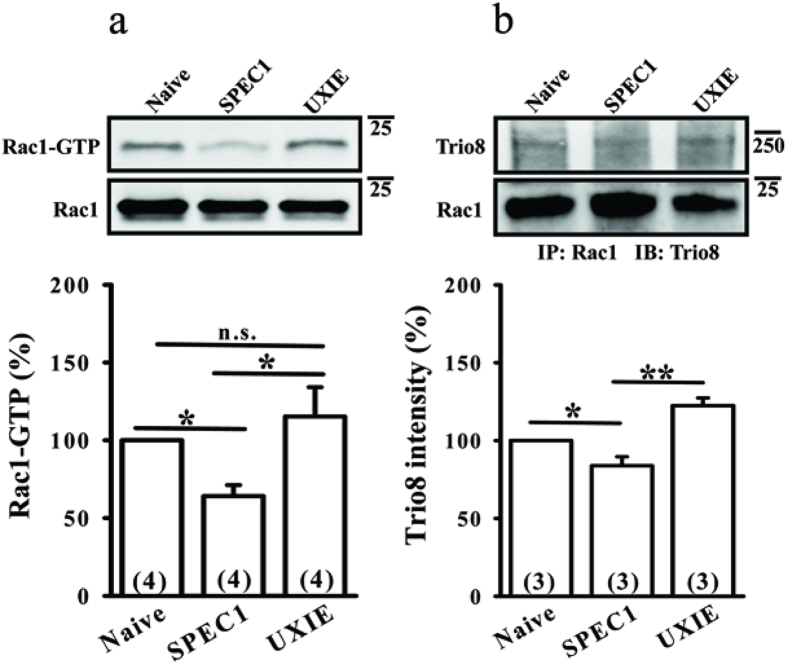
The SPEC-1 domain is important for SESTD1 to regulate Rac1 activity. (**a**) Representative immunoblots and corresponding densitometric analysis showing the levels of Rac1-GTP in naive, UXIE vector- or SPEC1-transfected neurons at 17 DIV. (**b**) Representative immunoblots and corresponding densitometric analysis showing transduced SPEC1 significantly reduced the interaction between Rac1 and Trio8 in hippocampal neurons at 17 DIV. *p < 0.05, **p < 0.01; ANOVA with Bonferroni’s *post hoc* analyses from four (**a**) or three (**b**) independent experiments. n.s., not significant.

To further confirm that the SPEC1 domain of SESTD1 is important for its regulation of Rac1 activity, we transduced hippocampal neurons with SPEC1 at 12–13 DIV and examined the Rac1-GTP at 17 DIV. We found that transduction of the neurons with SPEC1 resulted in a significant reduction of Rac1 activity (p < 0.05 compared with naïve or UXIE; Fig. 6a). In addition, transduced SPEC1 decreased the binding of Rac1 to Trio8 (p < 0.05 compared with naive and P < 0.01 compared with UXIE; Fig. 6b).

### Trio8 rescues dendritic spine loss in SESTD1-overexpressed neurons

**Figure 7:**
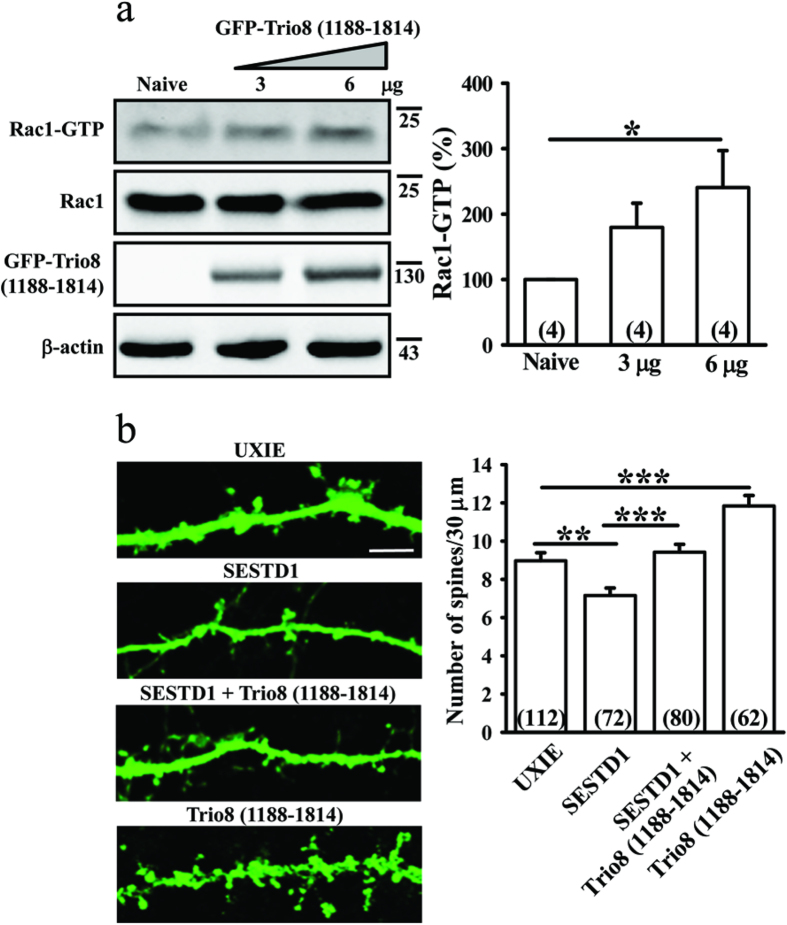
Overexpression of Trio8 (1188–1814) rescues SESTD1-induced decreases in Rac1 activity and dendritic spine density. (**a**) Representative immunoblots and corresponding densitometric analysis showing the levels of Rac1-GTP in naive and Trio8 (1188–1814)-transduced HEK293 cells. Note that overexpression of Trio8 (1188–1814) significantly increased Rac1 activity in a dose-dependent manner. (**b**) Representative images of hippocampal neurons transduced with UXIE vector, SESTD1, SESTD1 plus Trio8 (1188–1814) or Trio8 (1188–1814) at 12–13 DIV. Cells were fixed at 17 DIV and stained for GFP. The bar graphs show the quantification of density of dendritic spine (number/30 μm) of the representative groups. Scale bar: 5 μm. Numbers indicate the number of dendrites quantified. Statistical difference was compared with naive or UXIE vector groups, respectively. **p < 0.01, ***p < 0.001; ANOVA with Bonferroni’s *post hoc* analyses.

We also assessed the effects of Trio8 (1188–1814) expression on SESTD1-mediated decreases in Rac1 activation and dendritic spine density. We found that expression of GFP-tagged Trio8 (1188–1814) in HEK293 cells dose-dependently increases the amount of Rac1-GTP compared with naive group (F_(2,9)_ = 8.6, p < 0.01; Fig. 7a). Moreover, expression of Trio8 (1188–1814) significantly increased dendritic spine density (F_(1,173)_ = 16.7, p < 0.001) and rescued the effect of SESTD1 on dendritic spine density (F_(1,151)_ = 13.1, p < 0.001) of hippocampal neurons (Fig. 7b). These results suggest that SESTD1 limited dendritic spine density through interfering with the recruitment of Trio8 to Rac1, which in turn reduces Rac1 activation.

### SESTD1 decreases excitatory synaptic transmission

**Figure 8:**
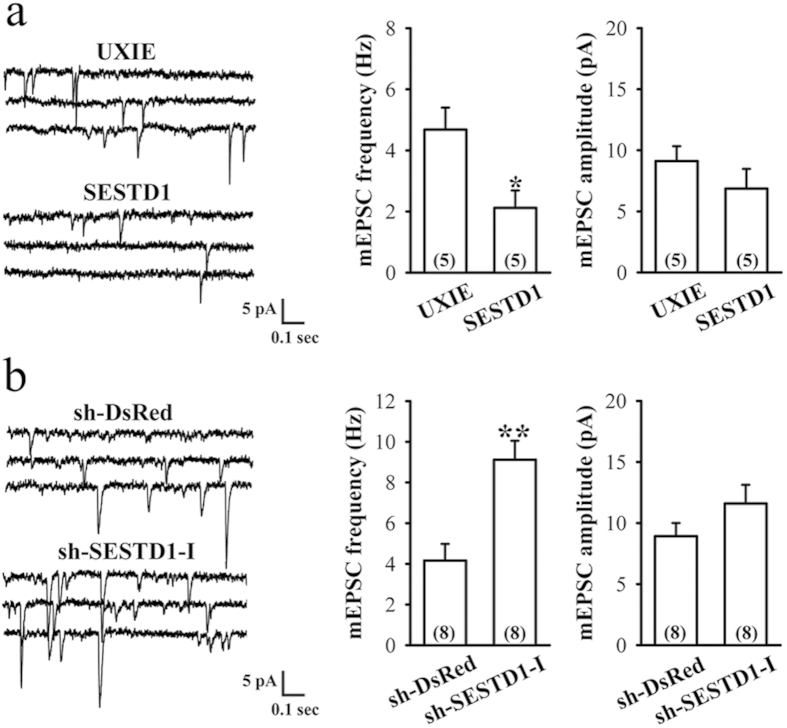
SESTD1 regulates mEPSCs in cultured hippocampal neurons. (**a**) Representative traces of mEPSCs were recorded in UXIE vector- or SESTD1-transduced neurons at 17 DIV. The bar graphs show the summary data of the average frequency and amplitude of mEPSCs in UXIE vector- or SESTD1-transduced neurons. (**b**) Representative traces of mEPSCs were recorded in sh-DsRed- or sh-SESTD1-I-transduced neurons at 17 DIV. The bar graphs show the summary data of the average frequency and amplitude of mEPSCs in sh-DsRed- or sh-SESTD1-I-transduced neurons. Numbers indicate the number of neurons analyzed. Statistical difference was compared with UXIE vector or sh-DsRed group. *p < 0.05, **p < 0.01; unpaired Student’s *t* test.

Changes in the density of dendritic spine are often coincided with changes in the density of excitatory synapses^7^. To examine the physiological significance of the SESTD1-mediated decrease in dendritic spine density, we recorded mEPSCs from hippocampal neurons overexpressing or knocking down SESTD1. As shown in Fig. 8a, overexpressed SESTD1 led to a significant decrease in the frequency of mEPSCs (2.12 ± 0.59 Hz, n = 5; t_(8)_ = 2.75, p = 0.03) compared with control UXIE vector-transduced neurons (4.68 ± 0.72 Hz, n = 5), whereas the amplitude of mEPSCs was not significantly affected (UXIE: 9.12 ± 1.21 pA, n = 5; SESTD1: 6.86 ± 1.62 pA, n = 5; t_(8)_ = 1.11, p = 0.29). In contrast, the frequency (sh-DsRed: 4.16 ± 0.82 Hz, n = 8; sh-SESTD1-I: 9.16 ± 0.93 Hz, n = 8; t_(14)_ = 4.0, p < 0.01) but not the amplitude of mEPSCs (sh-DsRed: 8.92 ± 1.08 pA, n = 8; sh-SESTD1-I: 11.61 ± 1.53 pA, n = 8; t_(14)_ = 1.43, p = 0.17) in neurons transduced with sh-SESTD1-I was significantly increased compared with control sh-DsRed-transfected neurons (Fig. 8b). These findings suggest that SESTD1 acts as a negative regulator on excitatory synaptic transmission in hippocampal neurons.

## Discussion

The morphology and density of dendritic spines are dynamically regulated by a variety of extracellular factors, including neurotrophic factors, hormones and neurotransmitters^27^,^28^. The cellular and molecular mechanisms that promote the formation of dendritic spines have been extensively studied, but relatively little is known about the factors that limit dendritic spine formation. In this study, we identify SESTD1 as a novel postsynaptic density protein that is colocalized with PSD-95 and plays a crucial role in negatively regulating dendritic spine density in hippocampal neurons. Our findings reveal a concerted mechanism through which SESTD1 binds specifically to Trio8 via its SPEC1 domain, thereby preventing the recruitment of Rac1 to the GEF domain of Trio8, resulting in reduced Rac1 activity and consequently decreasing dendritic spine density. Furthermore, we show that overexpressed SESTD1 resulted in decreased mEPSC frequency, revealing its negative regulatory role in excitatory synapse formation and function.

To our knowledge, this is the first study to demonstrate the presence and distribution of SESTD1 in hippocampal neurons. Specifically, we found that expression of SESTD1 protein is high at late embryonic stage (E18) and maintained at slightly decreased levels throughout postnatal brain development. While SESTD1 is widely distributed within neurons, it is abundantly expressed in the PSD of excitatory synapses. In addition to its ability to modulate TRPC4 and TRPC5 channel activity^18^, our data reveal a functional role of SESTD1 in modulating dendritic spine dynamics in neurons. We show that shRNA knockdown of SESTD1 increased, whereas overexpression decreased, dendritic spine density in hippocampal neurons. Intriguingly, we unexpectedly observed that although overexpressed SESTD1 resulted in a decrease in the number of filopodia, knockdown of endogenous of SESTD1 also caused a reduction in filopodia number in developing neurons. Given that filopodia are known to serve as precursors for spines^29^,^30^ and knockdown of SESTD1 resulted in an increase in the relative amounts of mature spines, it is likely that the observed reduction in filopodia number after SESTD1 knockdown is caused by promoting the filopodia-to-spine transition processes, which may mask the increase in filopodia number. These results suggest that SESTD1 has a negative regulatory role in dendritic spine formation.

How does SESTD1 reduce dendritic spine density? One mechanism by which it achieves this is through interference with spine formation. Dendritic spines show highly dynamic changes in density and morphology during neuronal development, as well as in the adult brain in response to various kinds of stimuli^3^. The Rho family GTPases, notably RhoA, Rac and Cdc42, are key regulators of dendritic spine formation and maintenance^5^,^16^. Indeed, it has been shown that inhibition of Rac1 activity decreases spine density, whereas inhibition of RhoA activity increases spine density and length^17^. Three classes of regulatory proteins are known to be involved in the regulation of Rac1 activity, including guanine dissociation inhibitors, GEFs and GTPase-activation proteins^31^. In this study, we demonstrate that blocking GEF-mediated Rac1 activation is responsible for SESTD1’s effect on dendritic spine density. Although SESTD1 shows moderate sequence conservation with Trio family members, it lacks a GEF domain and exhibits no GEF activity toward Rho family GTPases^19^,^20^. Instead, we show here that SESTD1 reduces Rac1 GTPase activity through interruption of Rac1-GEF Trio8 binding interaction. Rac1 GTPase activation is under tight and balanced regulation, mainly by GEFs of the Db1 family. Trio8 is a Db1 family protein, which has one GEF domain^32^. The activation of Rac1 by the GEF domain of Trio8 has been characterized in COS-7 cells^21^. Consistent with these findings, our data show that overexpression of Trio8 resulted in a dose-dependent increase in Rac1 activity in HEK293 cells (Fig. 7a). These results, together with our data indicating that overexpression of Trio8 essentially rescued the effect of SESTD1 on dendritic spine density, strongly suggest an important role for Rac1-Trio8 signaling inhibition in mediating the effect of SESTD1 on dendritic spine density.

Considering that dendritic spine density is the net result of spine formation and elimination, another possibility is that SESTD1 may promote spine elimination that leads to a decrease in dendritic spine density. In fact, the basal Rac1 activity is not only necessary for the generation of new spines but also for the maintenance of existing spine structure^16^. Unfortunately, the static imaging technique used in this study does not allow us to examine the turnover rate of existing spines. Thus, further studies using dynamic imaging techniques are required to address this possibility.

We also observed that the SPEC1 domain of SESTD1 is involved in inhibiting the recruitment of Trio8 to activate Rac1. It has been reported previously that the SPEC1 domain of SESTD1 bound to TRPC4 and TRPC5^18^. Using coimmunoprecipitation assay, we confirmed that SESTD1 binds specifically to Trio8, thereby preventing the recruitment of Rac1 to Trio8. On the basis of these observations, we infer that SESTD1 may serve as a dominant-negative inhibitor of Trio8’s activity. Indeed, the similar inhibitory mechanisms have been reported in other Db1 family proteins^33^,^34^,^35^. Our data also demonstrate that the C-terminal domains of Trio8 are required for its interaction with SESTD1, as Trio8 (1188–1814) mimicking the effect of full length Trio8 to rescue dendritic spine loss in SESTD1-overexpressed neurons (Fig. 7b).

What are the functional consequences of SESTD1-mediated reduction in dendritic spine density? In this study, we report that the reduction in dendritic spine density is paralleled by a decrease in excitatory synaptic transmission, as reflected by decreased mEPSC frequency. Our data show that overexpression of SESTD1 results in a decrease in the frequency but not amplitude of mEPSCs, whereas SESTD1 knockdown increases mEPSC frequency. A decrease in the frequency of mEPSCs is classically interpreted as a decrease in the number of glutamatergic synaptic contacts and/or a presynaptic inhibition of glutamate release probability^36^. Because we observed a significant decrease in mature dendritic spines in neurons overexpressing SESTD1, it is most likely that the observed decrease in mEPSC frequency by SESTD1 is caused, at least in part, by the loss of functional excitatory synapses. The lack of effect of SESTD1 overexpression on mEPSC amplitude implies that its action on excitatory synaptic transmission is not mediated by a change in postsynaptic responsiveness to glutamate. Although at this point it remains to be seen whether such modulation might occur in hippocampal slices or intact animals, it is tempting to speculate that SESTD1-mediated reduction of dendritic spine density might ultimately lead to the functional downregulation of synaptic strength.

In conclusion, an appropriate level of Rac1 activity under basal conditions is critical for maintaining normal dendritic spine density and synaptic function^24^,^25^,^26^. Here, we provide strong evidence that SESTD1 functions as a negative Rac1 regulator. SESTD1 may inhibit basal Rac1 activity by preventing the recruitment of Rac1 to Trio8, thereby reducing dendritic spine density and excitatory synaptic transmission in hippocampal neurons. Future experiments will explore whether dysregulation of SESTD1 may make a causal contribution to the pathogenesis of certain brain disorders.

## Methods

### Materials

Rabbit polyclonal anti-SESTD1 antibody was purchased from ProSci (Flint Place Poway, CA). Monoclonal anti-PSD-95, anti-β-actin and anti-Rac1 antibodies, and Rac1 activation assay kit were purchased from Millipore (Temecula, CA). Rabbit polyclonal anti-GFP and anti-synaptophysin antibodies were purchased from Abcam (Cambridge, MA). Monoclonal anti-bassoon antibody was purchased from Stressgen Bioreagents (Victoria, BC, Canada). Alexa-Fluor-488-conjugated anti-rabbit IgG and Alexa-Fluor-568-conjugated anti-mouse IgG were purchased from Molecular Probes (Eugene, OR). Rabbit polyclonal anti-Trio8 antibody was raised against recombinant Trio8 protein and was generated by LTK Biolaboratories (Taipei, Taiwan). Poly-L-lysine and bovine trypsin were purchased from Sigma-Aldrich (St. Louis, MO). Neurobasal-A, B-27 supplement, penicillin-streptomycin, L-glutamine and Lipofectamine 2000 were purchased from Invitrogen (Carlsbad, CA). T4 DNA ligation kit and restriction enzymes were purchased from New England Biolabs (UK). RIPA buffer solution was purchased from Thermo scientific (Rockford, IL).

### Establishment and maintenance of hippocampal neuron culture

All experimental procedures were approved by the Institutional Animal Care and Use Committee at the National Cheng Kung University, in accordance with National Institute of Health guidelines. Primary cultures of hippocampal neurons were prepared from E18–E19 embryos Sprague-Dawley rats as described previously^26^,^37^. Pregnant dams were killed by decapitation on day 18 or 19 of gestation under isoflurane anesthesia, and their embryos were quickly removed and placed on ice. Hippocampi of embryos were dissected out under a stereomicroscope and placed in ice-cold Hanks’ balanced salt solution (HBSS, Life Technologies, Gaithersburg, MD). Tissues were enzymatically digested with 0.2% trypsin for 15 min at 37 °C. Cells were disaggregated by trituration and plated on poly-L-lysine–coated petri dishes or glass coverslips in Neurobasal-A medium containing B27 serum-free supplement, 0.5 mM L-glutamine and antibiotics (50 U/ml penicillin and 50 μg/ml streptomycin). Cultures were incubated at 37 °C under 5% CO_2_/95% air and 90% relative humidity. Half of the growth medium was replaced every three days.

### HEK293 cell culture

HEK293 cells were maintained in DMEM (Invitrogen, San Diego, CA) supplemented with 10% fetal bovine serum (Invitrogen) and transiently transfected with indicated plasmid constructs using calcium phosphate or lipofetamine 2000 (Invitrogen) according to the manufactur’s instruction.

### DNA constructs

Full-length SESTD1 (amino acids 1–696) cDNA was amplified, using rat hippocampal cDNA library as the template, by PCR and subcloned into the *Xho*I and *Sma*I restriction sites of the pCAG-EGFP-C1 vector [N-terminal GFP tag under the control of the CAG (cytomegalovirus β-actin enhancer) promoter]. Constructs encoding different fragments of SESTD1 were generated by using PCR with the following primer pairs: SEC14, forward (5′-CCGCTCGAGATGGAGGCCTCGGTGATATTGCC-3′) and reverse (5′-GGGATCCGTCCTTCTCCTGCTCATTCCCC-3′); SPEC1, forward (5′-CGCTCGAGCCCGAAAGGTCCGTGGATTTAAAC-3′) and reverse (5′-GGGATCCCTGCTACGTCTACGCACAACATC-3′); SPEC2, forward (5′-CCGCTCGAGCCAGCTGATGGAGCATCGATT-3′) and reverse (5′-GGAATTCGAATGAGCTGTGGCTAACTCCGTAG-3′). The PCR product was then digested and subcloned into the pCAG-EGFP-C1 vector between *Xho*I and *Sma*I sites.

To construct constitutively active Rac1 (Rac1-CA), the mutation Gln^61^ to Leu^61^ (Q61L) was introduced into GFP-Rac1 with PCR-based site-directed mutagenesis using the following primer pairs: forward (5′-TGGGACACAGCTGGACTGGAAGATTATGACAGA-3′) and reverse (5′-TCTGTCATAATCTTCCAGTCCAGCTGTGTCCCA-3′) as previously described^38^,^39^. The introduced mutation was confirmed by DNA sequencing.

To generate constructs encoding fragments of Trio8 [Trio8 (1–1187) and Trio8 (1188–1814)], the rat full-length Trio8 gene was PCR amplified using the following primer pairs: forward (5′-GTCGACCGCATGAAAGCTATGGATGTTTTGCC-3′) and reverse (5′-GGAATTCGAATGGAAAGGTAAGGAAACTGAGC-3′). The PCR product was then excised with the restriction enzymes *Sma*I and subcloned into the pCAG-EGFP-C1 vector.

For SESTD1 knockdown experiments, shRNAs were subcloned into pLVTHM vector. Two sequences of shRNA targeted at different regions of rat *sestd1* gene coding sequence (sh-SESTD1-I and sh-SESTD1-II) and one shRNA directed against DsRed (sh-DsRed) as negative control were designed as following: sh-SESTD1-I, GCTCTGATTAACAATGGAAGT (base pairs 532–552); sh-SESTD1-II, GAAAGGATGTAACTATTGAAA (base pairs 1361–1381); sh-DsRed, AGTTCCAGTACGGCTCCAA, predicted by the BLOCK-iT™ RNAi Designer.

In some experiments, to validate SESTD1 protein expression and the specificity of anti-SESTD1 antibody, a lentiviral vector (UXIE) for production of viral particles with bicistronic expression of transgenes and EGFP under the control of ubiquitin promoter and separated by an internal ribosomal entry site was used for generating the full-length SESTD1 as described previously^40^.

### Lentiviral particle production

Engineered self-inactivating recombinant lentiviral particles were used for overexpressing or silencing the SESTD1 gene in cultured hippocampal neurons. All viruses were produced by co-transfection of lentiviral DNA with two helper plasmids in HEK293T cells: vesicular stomatitis virus envelope glycoprotein (VSV-G) and Δ8.9^37^,^41^. Media containing recombinant lentiviruses were harvested twice at 48 and 96 h after transfection and ultracentrifuged to obtain concentrated stock of lentiviral particles. Pellets were resuspended by phosphate buffer solution with titers of 10^8^ ~10^9^ units/ml. To evaluate Rac1 activity or Rac1-Trio8 interaction, hippocampal neurons were infected with lentivirus at 12–13 DIV and cell lysates were collected at DIV 17 for immunoprecitation and Western blotting analysis.

### Transient transduction of hippocampal neuronal cultures

For quantitative analysis of dendritic spine and filopodia number, hippocampal neuronal cultures (2 × 10^5^ cells) were grown on glass coverslips (10-mm diameter) inserted in 12 well. The neuronal cultures at 12–13 DIV were co-transfected with 2 μg EGFP-β-actin plasmid and 2 μg GFP-SESTD1, GFP-SEC14, GFP-SPEC1, GFP-SPEC2, UXIE, SESTD1, Rac1-CA, GFP-Trio8 (1188–1814), sh-SESTD1-I or sh-DsRed plasmid by Lipofectamine 2000 (Invitrogen) according to the manufacturer’s instructions and determined at 17 DIV. The efficiency of co-transfection can be close to 100% as previously described^40^,^42^.

### Preparation of subcellular fractions

Subcellular fractions were prepared according to the procedure of Huttner *et al.*^22^. The homogenate (H) was centrifuged at 1,000 × g for 10 min to remove nuclei and cell debris, and the supernatant (S1) was transferred to a new tube and centrifuged at 10,000 × g for 30 min to generate a crude synaptosomal fraction (P2). The supernatant (S2) was collected and spun at 165,000 × g for 2 h to yield a cytosolic fraction (S3) and a light membrane/microsome-enriched fraction (P3). The P2 fraction was washed once in HBS buffer [in mM: 10 HEPES-KOH, 142 NaCl, 2.4 KCl, 1 MgCl_2_, 5 glucose, 0.1 EGTA and 0.3 phenylmethylsulfonyl fluoride (PMSF); pH 7.5] and centrifuged once more at 13,000 × g for 15 min. It was then lysed in 10 volume of ice-cold H_2_O containing 0.3 mM PMSF for 30 min, buffered with 1 M HEPES-KOH, pH 7.4, and centrifuged at 25,000 × g for 30 min to generate the synaptosomal membrane fraction (LP1). The LP1 pellet was resuspended in lysis buffer containing 1% SDS and was centrifuged at 165,000 × g for 2 h to obtain the synaptic vesicle fraction (LP2) and soluble fraction (LS2). To obtain PSD fractions, the crude synaptosomal (P2) fraction was extracted with detergents, once with Triton X-100 (PSDI), twice with Triton X-100 (PSDII), and once with Triton X-100 and once with Sarkosyl (PSDIII). All procedures were performed at 4 °C.

### Western blotting analysis

Hippocampal tissue samples or cultured neurons were lysed in ice-cold Tris-HCl buffer solution (TBS; pH 7.4) containing protein phosphatase and protease inhibitors (50 mM Tris-HCl, 100 mM NaCl, 15 mM sodium pyrophosphate, 50 mM sodium fluoride, 1 mM sodium orthovanadate, 5 mM EGTA, 5 mM EDTA, 1 mM phenylmethylsulfonyl fluoride, 1 mM microcystin-LR, 1 mM okadaic acid, 0.5% Triton X-100, 2 mM benzamidine, 60 mg/ml aprotinin, and 60 mg/ml leupeptin). HEK293 cells were scraped and lysed in RIPA buffer solution with protein phosphatase and protease inhibitors. Samples were sonicated and spun down at 15,000 × g at 4 °C for 15 min. The supernatant was then assayed for total protein concentration using Bio-Rad Bradford Protein Assay Kit (Hercules, CA). Each sample was separated by electrophoresis in 7.5%, 10% or 4–12% SDS-PAGE gradient gels. Following transfer onto nitrocellulose membranes, blots were blocked in TBS containing 3% bovine serum albumin and 0.1% Tween 20 for 1 h and then blotted for 12–16 h at 4 °C with antibodies that recognized SESTD1 (1:3000), β-actin (1:2000), PSD-95 (1:2000), synaptophysin (1:2000), GFP (1:4000), Rac1 (1:2000) or Trio8 (1:2000), respectively. Each blot was probed with horseradish peroxidase-conjugated secondary antibody for 1 h and developed using the ECL immunoblotting detection system (GE Healthcare). Immunoblots were analyzed by densitometry using Bio-profil BioLight PC software.

### Immunoprecitation

Cells were dissolved in ice-cold TBS or RIPA buffer solution as described above. Cell lysates (300 ~ 500 μg protein) from different treatments were incubated with anti-Rac1 (1:300) or anti-SESTD1 antibody (1:200) in TBS overnight at 4 °C. The antibody-protein complexes were then pelleted with protein A-Sepharose beads. The complex was isolated by centrifugation and washed three times with TBS. Proteins eluted from the beads were subjected to 4–12% SDS-PAGE gradient gels and immunoblotting for anti-Trio8 (1:2000) or anti-GFP antibody (1:4000). Blots were stripped and reprobed with antibodies against Rac1 or SESTD1 to ensure equal amounts of proteins in each pull-down.

### Rac1 activation assays

The activity of Rac1 was determined by a Rac1 activation assay kit (Millipore) according to the manufactur’s instruction as previously described (Lee *et al.*, 2011). Briefly, Rac1-GTP from various treated lysates were pull-down using the GST fusion protein corresponding to p21 binding domain (PBD) of Rac1 (GST-Rac1-PBD) bond to agarose beads for 12 h at 4 °C. The beads were washed with ice-cold lysis buffer and resuspended in SDS sample buffer. Subsequently, the beads were washed four times with washing buffer, boiled in sample buffer, and separated on 4–12% SDS-PAGE gradient gels followed by immunoblotting with anti-Rac1 antibody to detect the presence of Rac1-GTP.

### Immunofluorescence staining

Hippocampal neurons were fixed with 4% paraformaldehyde in PBS containing 10% sucrose for 20 min at room temperature. After washing, neurons were permeabilized and blocked simultaneously in a solution containing 2% goat serum, 3% BSA and 0.2% Triton X-100 in PBS for 1 h at room temperature. The primary antibodies, anti-SESTD1 (1:1000), anti-PSD-95 (1:3000) or anti-bassoon (1:2000), were added in PBS containing 3% BSA and incubated overnight at 4 °C. After rinses with PBS, coverslips were incubated with appropriate Alexa-Fluor-488-conjugated and Alexa-Fluor-568-conjugated secondary antibodies for 1–2 h at room temperature, rinsed extensively in PBS, and mounted with ProLong Anti-fade media (Molecular Probes, Eugene, OR).

### Image acquisition and quantification

Images of neurons were obtained using a standard fluorescent microscope (BX51; Olympus, Tokyo, Japan) or a FluoView 1000 confocal microscope (Olympus) with sequential acquisition setting at a resolution of 1024 × 1024 pixels and producing images stacks with an average *z* depth of 2 ~ 5 μm. For higher magnification pictures, an Olympus Plan Apochromat 60× oil immersion objective was used. Transfected neurons with more than three dendrites longer than 50 μm were chosen randomly for quantification. Spine density was calculated by quantifying the number of spines on 15 ~ 25 neurons (~2–6 dendrites per neuron) for each condition. The number of dendrites used for quantification is indicated in the figures. Morphometric measurements were performed using MetaMorph image analysis software (version 6.3, Molecular Devices, Downingtown, PA). To quantify dendritic spine morphology, a ~30 μm portion of dendritic segment running distally from the cell body was used, and each individual spine present on the dendrites was manually traced. Filopodia was defined as dendritic thin protrusions >4 μm in length without a legible bulbous head. Spines including thin-, stubby- and mushroom-type were quantified. Thin spines were defined as thin dendritic protrusions <4 μm in length without a head. Stubby spines were defined as dendritic protrusions <4 μm in length without a spine neck. Mushroom spines were defined as dendritic protrusions <4 μm with a neck and legible bulbous head^26^,^42^,^43^. Blind conditions were used for the morphometric quantification of acquired images.

### Electrophysiological recordings

Whole-cell patch clamp recordings were made at room temperature from transfected and untransfected neurons at 17 DIV using the Axopatch 200B amplifier (Molecular Devices, Sunnyvale, CA) as previously described^26^,^37^. Transfected neurons were identified using standard epifluorescence. The recording chamber was continually perfused with 30–32 °C extracellular solution containing (in mM): 115 NaCl, 5 KCl, 2 CaCl_2_, 1 MgCl_2_, 5 HEPES, 20 glucose (pH 7.4), as well as tetrodotoxin (1 μM) to block action potential-dependent release of synaptic vesicles and bicuculline methiodide (20 μM) to block GABA_A_ receptor-mediated inhibitory synaptic transmission. Patch pipettes were pulled from thick-walled borosilicate glass and heat polished. The electrode resistance was typically 4–5 MΩ. The composition of internal solution was (in mM): 116 K-gluconate, 6 KCl, 2 NaCl, 20 HEPES, 0.5 EGTA, 2 Na_2_ATP, 0.3 Na_3_GTP, 5 QX-314, which had an osmolarity of 290–295 mOsm and pH of 7.3. Neurons were voltage clamped at −60 mV to record mEPSCs. Electrical signals were low-pass filtered at 2 kHz, digitized at 10 kHz using a 12 bit analog-to-digital converter (Digidata 1320, Molecular Devices). An Intel Pentium-based computer with pCLAMP software (Version 8.0; Molecular Devices) and Mini Analysis 4.3 (Synaptosoft, Leonia, NJ) were used for on-line acquisition and off-line analysis of the data. The detection threshold was set at 3 pA, and neurons with noisy baselines were discarded. To be included for analysis, mEPSCs had to have a monotonic rising phase with a rise time of <6 ms and decays that followed an exponential time course with a decay time constant of <25 ms. Frequencies of mEPSCs were calculated by dividing the total number of detected events by the total time sampled.

### Statistical analysis

Data for each experiment were normalized relative to controls and presented as mean ± s.e.m. The significance of any difference between means was calculated by using ANOVA with Bonferroni’s *post hoc* analyses in comparison two groups within multiple groups or the unpaired Student’s *t* test between two groups. Probability values (p) less than 0.05 were considered to be statistically significant.

## Additional Information

**How to cite this article**: Lee, C.-C. *et al.* The phospholipid-binding protein SESTD1 negatively regulates dendritic spine density by interfering with Rac1-Trio8 signaling pathway. *Sci. Rep.* **5**, 13250; doi: 10.1038/srep13250 (2015).
